# Editorial: The Interface between Psychoanalysis and Neuroscience: the State of the Art

**DOI:** 10.3389/fnhum.2020.00199

**Published:** 2020-06-03

**Authors:** Massimo di Giannantonio, Georg Northoff, Anatolia Salone

**Affiliations:** ^1^Department of Neuroscience, Imaging and Clinical Sciences, G. D'Annunzio University, Chieti, Italy; ^2^University of Ottawa Institute of Mental Health Research, Ottawa, ON, Canada; ^3^Brain and Mind Research Institute, University of Ottawa, Ottawa, ON, Canada

**Keywords:** psychoanalysis, neuroscience, neuropsychoanalysis, neuroimaging, psychodynamic psychotherapy

The Research Topic “The Interface Between Psychoanalysis and Neuroscience: The State of the Art” was cross-linked in two Frontiers journals: *Frontiers in Human Neuroscience* and *Frontiers in Psychology* and the contributing authors could choose to submit their article to the journal that best suited the research content. Among the 11 contributions finally accepted, 7 have been published in *Frontiers in Human Neuroscience* and the other 4 in *Frontiers in Psychology*. This balanced distribution between the two journals is in line with the wide range of topics dealt with by the various authors, from the theoretical reviews aiming at converging psychoanalytic concepts with discoveries in neuroscience, to the neuroscientific investigation of the mechanisms involved in psychodynamic psychotherapy.

The Research Topic includes articles presented in the form of Review, Hypothesis and Theory, Perspective Article, and Original Research Article.

The implicit question posed to the contributors was to offer their point of view on the usefulness of combining a theoretical psychoanalytic approach with an empirical-experimental one derived from neuroscientific studies. The idea was to encourage an overall theoretical expansion capable of promoting a more complex vision of the mental and psychic functioning underlying scientific research. A validation of psychoanalytic theories has not been deliberately indicated as the purpose of the Research Topic, in favor of a possible and bidirectional conjugation of the same theories with empirical evidence, with the belief that a conceptual enlargement of psychic and cerebral functioning could be an indispensable necessity both for psychoanalysis and for neuroscience. As expected, the authors answered the question starting from different perspectives. In papers of a more theoretical nature, some authors have placed psychoanalytic concepts at the basis of neuroscientific reflection, whereas others applied a psychodynamic theory of mind to benefit the interpretation of empirical neuroscientific evidence. Still other authors have designed experimental studies aimed at investigating psychic mechanisms that were not explicitly conceived according to a psychoanalytic theoretical framework, but that paved the way for a re-evaluation and reconceptualization of psychoanalytic concepts themselves. Finally, for some, these mixed aspects represented the starting point for implementing therapeutic approaches in specific mental disorders.

In particular, several authors have used a framework of the discoveries that derive from Affective Neuroscience: one paper investigated the instinctive component of the imagination, combining cardinal concepts of psychoanalytic theory (dynamic mental structures such as Unconscious Fantasies) with the function of the basic emotional-motivational systems, especially the SEEKING SYSTEM, in a neuro-ethological context, possibly leading to perspective changes in psychology and psychotherapy (Alcaro and Carta); another article highlighted different components of aggression, especially the Dominance motivational/emotional system, at the basis of competitive behaviors in human relationships, often compromised in different mental disorders (Giacolini and Sabatello).

Another neuroscientific model, based on the centrality of the Default Mode Network and Cortical Midline Structures in the expression of Self, was used in relation to a psychodynamic perspective, with consequent, important clinical and therapeutic implications. Some authors conceived multiple levels of the Self (relational alignment, self-constitution, self-manifestation, self-expansion), corresponding to different levels of personality organization, theorizing for the first time an integrated neurobiological and clinical personality model in which spontaneous brain activity plays a fundamental role (Scalabrini et al.). According to other authors, a similar neuropsychodynamic perspective could be useful to conceive an imbalance between the Default Mode Network and the Executive Network as a core feature in depressive disorders, hypothesizing therapeutic interventions aimed at contrasting this imbalance by acting on specific mechanisms of compensation and defense (Boeker and Kraehenmann).

Focusing on unresolved trauma in mothers, a neuroscientific perspective of the Attachment Theory (in particular, the Dynamic Maturational Model of Attachment and Adaptation—DMM) is used to investigate the reorganization of their attachment style and the neurobiological correlates underlying the behavioral response of adaptation to motherhood (Iyengar et al.).

The experience of pleasure, starting from classic psychoanalytic concepts of instinct and affect, is conceptually enriched in the light of studies on primary-process emotional feelings with a consequent reflection on libido-independent intrinsic motivations and their role in structuring self-related processes (Moccia et al.).

Among the authors who promote the importance of the link between psychoanalytic theories and neuroscientific investigations for therapeutic approaches for specific psychic disorders, the proposal of emotional regulation as a central ability favoring a balanced defensive structure in healthy psychological functioning stands out (Frederickson et al.).

Also the interrelation between a theoretical approach based on Embodied Cognition studies and classical psychoanalytic theory frames the therapeutic proposal of authors who suggest the importance of psychoanalytic psychodrama for the treatment of internet addiction disorder and eating disorders, which is proposed to stimulate a recovery of the Embodied Self with respect to the excessive Narrative Self (Scorolli et al.).

Moreover, a reflection on improving the specificity of therapeutic approaches is presented in the context of pharmacotherapy and its psychic meaning within a psychoanalytical treatment (Iannitelli et al.).

Finally, with a focus on unconscious processes, two Original Research Articles propose interesting results both on the unconscious influence of phonological ambiguity detection in response to subliminal stimuli, correlated with the defensive personality structure of the subject (Bazan et al.) and on the effects of 3-Hz binaural beat stimulation on sleep stages (Jirakittayakorn and Wongsawat).

In addition to the satisfactory number of published articles, our Research Topic has garnered considerable interest among readers. In our opinion, this represents the signal of a growing interest in the area of dialogue between psychoanalysis and neuroscience, the response to the need of both disciplines to broaden their horizons in the direction of the complexity of the human being, in which a separation between psychic and neurobiological functioning can no longer be hypothesized. Psychoanalysis represents a most complex theoretical model of the human mind and could be a useful stimulus for neuroscientific research oriented toward the subjectivity and the relational nature of human experience, and the importance of unconscious processes. On the other hand, while remaining two distinct areas, adequately designed neuroscientific research can contribute to an expansion of the metapsychological perspectives of psychoanalysis itself.

Much work certainly remains to be done and the present Research Topic is not representative of all the areas of interest currently investigated in neuropsychoanalysis; yet, in our opinion it represents a fruitful stimulus to move forward in a definitely constructive direction.

## Author Contributions

All authors listed have made a substantial, direct and intellectual contribution to the work, and approved it for publication.

## Conflict of Interest

The authors declare that the research was conducted in the absence of any commercial or financial relationships that could be construed as a potential conflict of interest.

